# Application of precision livestock farming: challenges and opportunities

**DOI:** 10.5713/ab.250895

**Published:** 2026-03-11

**Authors:** Muhammad Ikhsan Sani, Mariska van der Voort, Bedir Tekinerdogan, Henk Hogeveen

**Affiliations:** 1 Business Economics Group, Wageningen University and Research, Wageningen, The Netherlands; 2 Computer Technology, School of Applied Science, Telkom University, Bandung, Indonesia; 3 Information Technology Group, Wageningen University and Research, Wageningen, The Netherlands

**Keywords:** Mastitis, Precision Dairy Farming, Precision Livestock Farming, Reproduction, Sensor System

## Abstract

Sensor systems have increasingly been explored as tools to support precision livestock farming, particularly in monitoring cow health and improving decision-making. This systematic literature review aims to evaluate advancements in sensor systems for detecting health conditions in dairy cows especially on mastitis, fertility, locomotion, and metabolic disorders. Relevant articles published between 2014 and 2024 were identified from Scopus. Each article was categorized by health condition and assigned to one of four development levels: sensor technique (Level I), data interpretation (Level II), integration of information (Level III), and decision making (Level IV). Relevant information from the articles was systematically reviewed and discussed. We identified 132 articles published in the past 10 years, describing a total of 151 sensor systems. Most sensor systems were aimed at mastitis and reproduction, followed by locomotion and metabolic disorders. The far majority of the articles were at level II (data interpretation) presenting research on (novel) algorithms to detect disease. A large number of different statistical, machine-learning or deep-learning models were described and evaluated, among others random forests. Level II systems applied statistical analysis or machine-learning/deep-learning models (e.g., random forests, you only look once, support vector machine, or convolutional neural network). These algorithms used a wide range of sensor data. Only a few articles aimed at level III research, integration of information and decision support. The Level III sensor systems integrated information from the sensor with economic information and other information (i.e., medication dosage, cost per disease, and supplier selection) and simulated various treatment scenarios. This review highlights the need for sensor systems research to be driven by real-world requirements for on-farm decision making. To move from proof-of-concept toward practical, future research must integrate sensor outputs with herd records and financial models, validate systems across multiple farms and at higher data frequencies, and embed economic evaluation alongside sensitivity and specificity metrics. Addressing these technical, integration, and economic challenges is essential before sensor systems can fully support automated, value-driven health management on commercial dairy farms.

## INTRODUCTION

Precision livestock farming (PLF) has emerged as a concept through advanced technologies aimed at automatic, real-time monitoring of animal production, animal health and welfare, and environmental impact [1]. Because of the relatively low number of animals per farm and the high value of individual animals, within PLF a specific concept has developed known as precision dairy farming (PDF). PDF focuses on integrating sensor technologies to improve on-farm decision-making, particularly in managing dairy cow health and productivity [2]. Health-related PDF research often emphasizes mastitis, lameness, fertility issues, and metabolic disorders. Data form the basis for PDF and within the field and these data are often collected by sensors. Sensors for PDF can be classified into two categories: attached and nonattached. Attached sensors can be categorized as on-cow sensors, which are mounted externally to the cow’s body, or in-cow sensors, which are implanted inside (e.g., rumen bolus or implant). Nonattached sensors are off-cow sensors that cows pass by, over, or through for measurement. Because lactating dairy cows are milked daily, measuring milk offers very interesting opportunities. Measuring milk can be done by in-line sensors and online sensors. In-line sensors detect continuously within the milk from the cow, e.g., electrical conductivity (EC), milk flow, or somatic cell count (SCC). Online sensors automatically take a sample from the milk which is subsequently examined [3].

Within the PDF research field, an influential publication in 2013 proposed a widely adopted definition of sensor systems and a framework for classifying their contribution to decision-making [3]. In this work, a sensor system was described as the combination of a sensing device and the software that transforms raw measurements into meaningful information or actionable advice. In practice, such systems quantify physiological or behavioral parameters related to cow health or reproductive status, thereby enabling automated on-farm detection of condition changes that may require farmer intervention [3,4].

The same publication introduced a four-level framework to characterize sensor systems according to their function: (I) the technical measurement level, (II) data interpretation, (III) information integration, and (IV) decision support. A review of 126 studies revealed that most research had focused on level II, that is, developing algorithms to convert sensor data into the detection of health or estrus events. In contrast, little work has been done on integrating information across systems (level III) or evaluating the management and economic consequences of sensor-based decisions (level IV). The lack of economic assessments also highlighted limited knowledge about the cost-effectiveness of implementing such technologies. Overall, the review emphasized the importance of defining clear objectives regarding the type of health or behavioral information a sensor system should deliver and ensuring that this information can be translated into practical, actionable management decisions for farmers.

Despite the research efforts, real-world adoption of PDF remains limited [5,6]. Also in practice, most sensor systems operate at early development stages and often lack direct links to actionable decision support for farmers [7,8]. Four main reasons for adoption and non-adoption have been identified by Russell and Bewley [9] and Steeneveld et al [10], being: knowledge about available systems, insight into the economic benefits of investments, clarity and applicability of information provided by the sensor system, and expectations of future developments. There is a gap between technical development and practical implementation for on-farm decision support. Given the technological advancements since then, particularly with the emergence of machine learning (ML), big data analytics, and the Internet of Things (IoT), it can be expected that more recent research in PDF has progressed beyond technical validation to practical application and on-farm decision support [11,12]. Therefore, the objective of this review is to identify the state of dairy cow sensor systems between 2014 and 2024, assess their progression, and determine how far research has moved from technical validation toward practical, on-farm decision support.

## ARTICLE SELECTION

In this review, we adopted a systematic evidence review approach and followed the Preferred Reporting Items for Systematic Reviews and Meta-Analyses (PRISMA) guidelines to ensure rigor, transparency, and reproducibility [13,14]. The article search was conducted on the Scopus database using a specific search string (Table 1). We considered peer-reviewed journal articles and conference proceedings published between January 2014 and December 2024.

**Figure 1:**
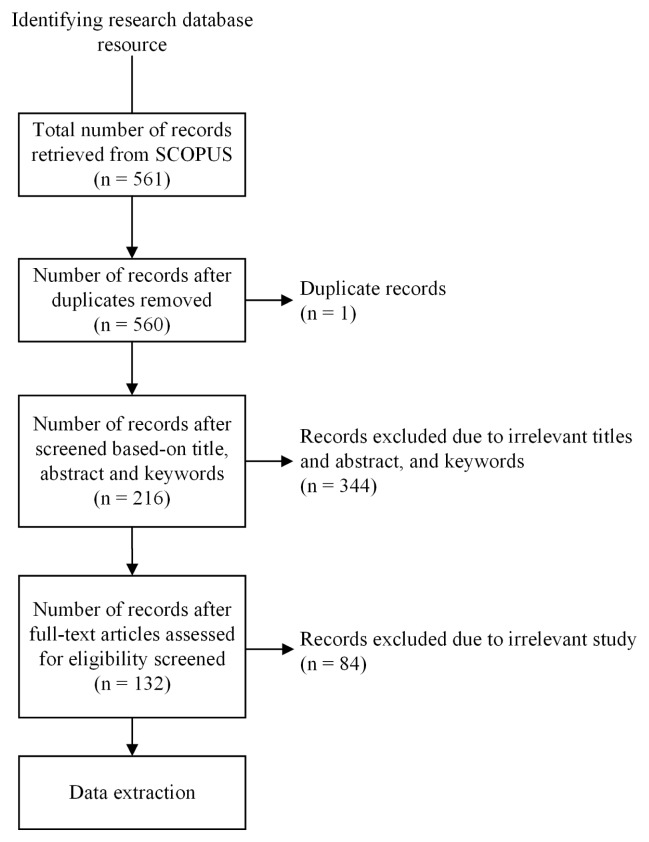
PRISMA flow diagram. PRISMA, Preferred Reporting Items for Systematic Reviews and Meta-Analyses.

The search strategy employed Boolean operators to refine keywords and ensure relevant articles were captured. Search terms included three groups of search terms: 1) terms associated with dairy farming; 2) terms associated with sensor systems and 3) terms associated with the specific dairy cattle disorders. These three groups were combined (Table 1). All retrieved records of articles were screened for English language and availability of full text. After excluding non-dairy topics (e.g., beef, pigs, poultry, and sheep), a total of 561 unique records were retrieved from Scopus on 7 April 2025. The PRISMA flowchart in (Figure 1) shows the results of the article selection process. This process screened a total of 561 records of articles. After removing duplicates, 560 records of articles remained for title and abstract screening. Titles and abstracts were screened for relevance and full-text article eligibility. Articles were included if they reported on the application of sensor systems in monitoring dairy cow health and addressed at least one of the health conditions mentioned (mastitis, fertility, locomotion, or metabolism). From these, 344 records of articles were excluded as irrelevant. The full texts of 216 articles were assessed for eligibility. At this stage, 84 additional records of articles were excluded for reasons such as insufficient methodological detail or focus on non-relevant species or health issues. As a result, 132 records of articles met all inclusion criteria and were carried forward for data extraction and analysis.

## REVIEW FRAMEWORK

**Figure 2:**
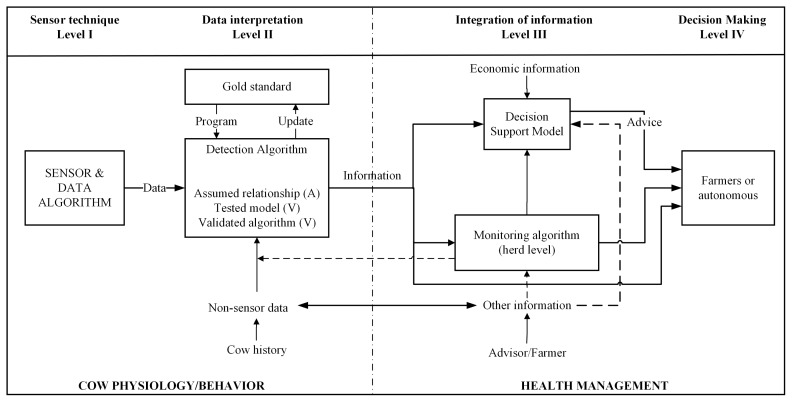
Framework of Rutten et al [3] on the levels of sensor information in dairy farm management. Adapted from Rutten et al [3] with CC-BY.

To categorize sensor systems on selected articles, the framework of Rutten et al [3] (Figure 2) was used. The framework describes the levels of sensor development, from the sensor technique (Level I) to the effects of implementing sensor-based decisions (Level IV). Each sensor system in the articles was classified according to the following characteristics:

• Level I sensor techniques: The sensor system on this level primarily measures an aspect of the dairy cows’ health condition.
• Level II data interpretation: The sensor system on this level interprets data and measures changes in the raw sensor data to produce information about the cows’ status. Some algorithms are involved in data processing for detecting, predicting or prescribing the potential health risks, such as changes in feeding behavior indicating diseases.
• Level III integration of information: The sensor system on this level integrates the sensor information with other information (such as economic information), to produce advice for the farmer. Furthermore, information of individual cows can be aggregated by a monitoring algorithm at the herd level. The output of this algorithm can be seen as either general information on the herd health for the farmer or additional data input for the detection algorithm.
• Level IV decision-making support and automation: The sensor system on this level not only detects health changes but also recommends or executes actions.

In addition to this classification, the following attributes were extracted for each sensor system, where available: sensor type, data algorithm, gold-standard definition and sampling frequency, non-sensor data sources, decision support model, sensitivity and specificity metrics, and final system output. For the evaluation of sensor system performance, this review focused on confusion matrix–based metrics, including sensitivity, specificity, and accuracy. These metrics were selected because they were most consistently reported across the included studies, allowing comparison across different health conditions and sensor systems. Although other performance measures such as the area under the curve (AUC), F1-score, or correlation coefficients can provide valuable additional insights, these metrics were reported inconsistently and were often not directly comparable across studies. As a result, they were not included in this review.

It is possible that commercially available or patented sensor systems have yet to be evaluated in peer-reviewed articles. Because this review focuses on systems that were published in journals or conferences between 2014 and 2024. Level IV systems can exist in the form of commercially available products or proprietary hardware which is not published in recent scientific journals or conferences. As a result, some farm-adopted technologies, especially new or region-specific solutions may be missing. For example, Dairy Brain project (Dairy Brain – The Next Big Leap in Dairy Farm Management Using Coordinated Data Ecosystems – UW–Madison) is one project that integrates Artificial Intelligence into the dairy farming operation for decision making. Another example is DairyMGT that has been published in 2012. It is a suite of 30 computerized decision support system or decision support tools aimed to assist dairy farm managers and dairy farm advisors to improve their continuous decision-making and problem-solving abilities [15].

Articles that describe an intervention or monitoring approach that relies on manual data entry, human-observed scoring without sensors are excluded. For example, visual locomotion scoring charts and management dashboards that accept only farmer entered health records do not meet our definition of an automated sensor system [16,17]. Also, devices that are designed primarily for production optimization (e.g., feed intake) but not for disease or health monitoring/detection have been excluded [18].

## SENSOR SYSTEMS

**Figure 3:**
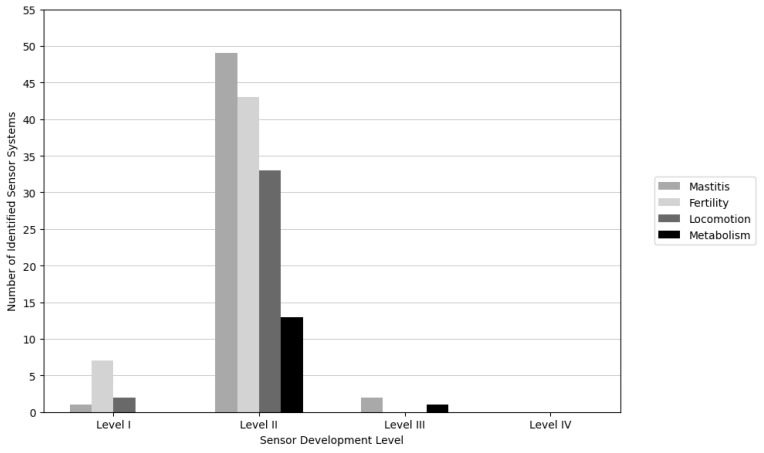
The number of identified sensor system’s on reviewed articles for mastitis, fertility, locomotion, and metabolism categorized by development level is as follows: Level I = technique, Level II = data interpretation, Level III = integration of information, and Level IV = decision making.

Figure 3 shows the categorization of articles that study sensor systems by development levels (I to IV) across four dairy cow health conditions. From the 132 selected articles that described 151 sensor systems, 52 systems (34.43%) addressed mastitis, 50 systems (33.11%) addressed fertility, 35 systems (23.17%) addressed locomotion, and 14 systems (14.12%) addressed metabolism. Also the number of articles describing fertility and locomotion issues was relatively large. In terms of sensor development levels, the far majority of the articles presented systems at level II (data interpretation). Only two articles that described three sensor systems at the level III (integration of information). No articles were found that described a system at level IV. In the next paragraphs, the sensor systems that monitor one or more of the four dairy cow health conditions are described and organized by their development level.

### Mastitis

A total of 52 sensor systems for detection of mastitis were identified. Table 2 shows the summary of the sensor systems that were used for mastitis detection.

#### Level I: sensor technique

For detection of mastitis, articles were identified, describing three sensor systems that collect only raw data. One described a cell-counting chamber and a miniature fluorescent microscope [19,20]. Two of the sensor systems utilized thermal imaging and infrared cameras as noninvasive tools to monitor udder health status in dairy cows [21,22].

#### Level II: data interpretation

At this level, 49 articles described the statistical or ML algorithms that were used in sensor systems to interpret raw sensor data into mastitis classifications and validate model predictions against clinical diagnoses or SCC thresholds (Table 2). For example, most articles used EC or SCC data in logistic regression and random forest models, while [23] applied thermal infrared imaging features in a classifier whose outputs were compared directly to inline SCC measurements as the gold standard verification set.

A wide range of sensitivities and specificities can be observed. Sensitivities vary from 26% to 98% and specificities vary from 40% to 99.8% (Table 2). The best combined performance was 82% to 95% sensitivity in combination with a specificity of 93% to 95% [24]. When specificity is seen as important in mastitis detection, a combination of a specificity of 99.8% with a sensitivity of 38% to 53% [25].

#### Level III: integration of information

One article described a sensor system at level III. The sensor system is capable of uploading and managing veterinary treatment records, extracting medication and diagnosis lists, performing statistical analysis of treatment data, predicting disease incidence, and calculating linear trends for disease data over various periods [26]. It also has capabilities to optimize cost management using predictive data, helping farmers to plan expenses and select suppliers.

### Fertility

A total of 50 sensor systems for detection of fertility condition were identified. Table 3 shows the summary of the sensor systems that were used for fertility detection.

#### Level I: sensor techniques

Nine articles described ten sensor systems (some articles described more than one sensor system). Two systems were described that monitor reproductive activity using a neck-collar device and a noseband sensor [27,28]. One system used a sound-based sensor to record estrus vocalizations in individual cows [27] and another system determined the precise time of ovulation using an ultrasound technique [29]. Two sensor systems used a 3D accelerometer to count steps and linked elevated step counts to estrus, assuming a direct relationship between activity and reproductive status [30,31]. Two systems used video cameras that were mounted above the barn to monitor dairy cows’ fertility-related behaviors [32,33]. Finally, one sensor system used a rumen bolus sensor for evaluation of dairy cow behavior after calving [33].

#### Level II: data interpretation

Three articles described sensor systems containing fertility detection models that were validated using an external dataset, other than the dataset that was used for creating the model [34,35,36]. The other 42 sensor systems at this level described a statistical or algorithmic link between sensor outputs and reproductive events (e.g., estrus, insemination success, or confirmed pregnancy). The majority used accelerometers or pedometers to detect estrus through elevated activity, often validating against timed artificial insemination records or ultrasound pregnancy checks. ML algorithms used in the sensor systems are random forests, logistic regression, perceptron, and support vector machines. Most articles were aimed at detecting estrus. In general, estrus detection results were very good, up to a sensitivity of 1 and specificities close to 1.

### Locomotion

A total of 35 sensor systems for detection of locomotion conditions were identified. Table 4 shows the summary of the sensor systems that were used for locomotion problem detection. The detailed description and system quality of each level for fertility detection are described below.

#### Level I: sensor techniques

Two articles describe two sensor systems that measured locomotion condition of dairy cows using different types of sensors. One system consists of a noseband halter with an integrated pressure detector to measure movement patterns [28]. The other system used wearable sensor to monitor dairy cows activity i.e. eating and ruminating [37].

#### Level II: data interpretation

Thirty-one sensor systems interpret sensor data (from accelerometers, force plates, or video) to detect or predict lameness indicators/locomotion score. One sensor system used accelerometer data from single sensors on the cows’ left flanks and compared its results with those obtained through classical ML applied to the same raw data [38]. One system describes the development of the model for automatic lameness detection using data from sensor systems, that were attached to the hind limbs (accelerometers) and the head (noseband sensors) [39]. One article describes a prediction model for lameness in dairy cattle using a combination of remote sensor technology and other animal records that will translate sensor data into easy to interpret classified locomotion information for the farmer [40]. One system integrates a temperature sensor, a global positioning system module, and a 3D accelerometer using eXtreme Gradient Boosting (XGBoost) and Random Forest classifiers to classify cattle activities [41].

Also here, it seems that classification results were relatively good, with sensitivities ranging from 58% to 97% and specificities ranging from 42% to 99%. The best performance was a sensitivity of 97% in combination with a specificity of 98% for a system aimed at distinguishing healthy vs. severely lame cows [42].

### Metabolism

A total of 14 sensor systems for detection of metabolism problems were identified. Table 5 shows the summary of the sensor systems that were used for metabolism problem detection.

#### Level II: data interpretation

A total of 14 sensor systems were categorized into level II. Regarding metabolic diseases, a wide range of sensor systems and problem identifiers were used. Systems were described to detect clinical ketosis, subclinical ketosis and metabolic problems in general. Moreover, the metabolic situation was in some articles also evaluated at the lactational level, rather than in detecting cases. Most articles used ML, supervised or unsupervised. One article used statistical evaluation and one article used [43,44] long short-term memory models (LSTM) [45]. Finally, a wide range of sensor systems or measures derived from sensor systems were used: rumen pH (rumen bolus), body temperature, milk composition (in-line milk analyzer), accelerometer data from neck collars or ear tags, rumination behavior (rumen bolus or noseband), body condition score (BCS; camera-based image analysis), rumination patterns, physical activity and lying (3D accelerometer), Fourier-transform infrared measurements and automated health alerts.

Most studies only looked at sensitivity and seemed to reach a decent sensitivity, ranging from 57% to 93%. Only one study evaluated both the sensitivity and specificity for metabolic disorders with a sensitivity of 71% in combination with a specificity of 47% [46].

#### Level III: integration of information

One article mentioned sensor system-incorporated dairy cow metabolism information and economic information [26]. The system calculates medication dosages and costs per disease, streamlines supplier selection, and simulates various treatment scenarios, thereby identifying high-cost diseases with potential savings.

## MAIN OBSERVATIONS

This review shows that sensor systems developed between 2014 and 2024 are mostly at Level II (91%), while only a few sensor systems (2%) reached Level III and none achieved Level IV. This indicates that research was still heavily focused on data-interpretation algorithms rather than on integration or decision-making functionality. Rutten et al [3] reported a similar distribution at Levels I and II, and none achieved Level III or IV systems. Given the progress in sensor technology and scientific developments in the past 10 years, we would have expected that much more of the research would be closer to the actual use of sensor systems, especially regarding mastitis and fertility, health issues that were already studied extensively when the Rutten et al [3] paper was published. It seems that research is repeating the type of studies that have been done for decades now, mostly working with the same type of sensors but especially trying to develop new algorithms to detect mastitis and estrus.

Although the objectives and level of research did not change much over time, the type of algorithm that was used did change. Rutten et al [3] found that the majority of algorithms were decision trees and neural networks. In this paper, we found that still some very basic algorithms were used, such as threshold rules and logistic regression. However, also many more recent algorithm such as you only look once (YOLO) or enhanced fusion MobileNetV3 you only look once (EFMYOLOv3), were described. Most of the articles aimed at developing detection algorithms with relatively new ML methods. These studies were motivated by the expectation that a different, more powerful ML method would improve the detection performance. However, it seems that the performance of the newer algorithms did not improve the detection performance very much. This leads to the question of whether newer and more powerful ML techniques will ever improve the detection performance, or whether the biological variations present in animals prevent us from detecting a disease such as clinical mastitis, or a reproductive status as estrus any better than we do now. This has been explained for mastitis by Hogeveen et al [47] and they proposed novel research, not into novel sensors or ML algorithm, but in a change of management, adapted to the power of sensor systems, rather than to focus on clinical mastitis alone. In other words: to adapt animal health management to the potential of sensor systems, rather than to adapt sensor systems to current health management. An example for such an approach is provided for lameness interventions, where the current approach of treating severe lame cows is replaced by sensor-based treatment of moderately lame cows based upon sensor systems [48].

The performance of detection of estrus is sufficient, because that is the type of sensor system which has been adopted the most. Although there are no routine statistics available on the adoption of sensor systems, a few studies have been published in the past. In the Netherlands, in 2013, 39% of the farmers had adopted sensor systems [49]. To the best of our knowledge, there is no more recent overview that has been provided. The majority of these adopters were using sensor systems associated with automatic milking, i.e., sensor systems for mastitis detection. At that time, only 12% of the farmers with a conventional milking system adopted a sensor system. Almost all these farmers were using 3D accelerometers to detect estrus. Likewise, in Switzerland in 2018, the adoption rate of sensor systems was 35%, also with a majority having automatic milking system [50]. In the same year, the adoption of technology in Ireland was only 8% [51]. The best adoption rate was found in a study in Italy with an adoption rate of 78% in 2020 [52]. Although not supported by any statistics, according to our knowledge and experience, the adoption of PDF other than automatic milking systems is lagging behind expectations that we once had.

## SENSOR PERFORMANCE

Across mastitis sensor systems, reported sensitivities ranged between 26% and 98% and specificities between 40% and 99.8%. According to the performance standards proposed by Hogeveen et al [47], only a small proportion of systems approach the thresholds required for reliable on-farm detection, particularly for severe CM (>95% sensitivity and >99% specificity). The wide variability in performance, often due to differences in validation protocols, sensor placement, and data windows, suggests that current systems remain insufficiently accurate for autonomous decision-making. Most systems risk false-positive alerts, which could undermine farmer trust and hinder adoption. Moreover, farmers need more than accurate alerts or predictive models. They need integrated dashboards that merge sensor data with treatment options and the expected economic value of each option. That way creating automated workflows that trigger actions. Researchers should partner with veterinarians and animal scientists to develop interventions in line with the information that sensor systems provide. This is a challenge, especially when we start working with interventions that were not possible based upon the current disease detection (by the farm workers).

The reported sensitivity of fertility detection systems ranged between 45% and 98%, with specificity varying between 70% and 99% depending on the sensor type and validation method. Not all sensor systems reported sensitivity, specificity, and accuracy. Behavior-based sensor systems such as activity, rumination, or vocalization, showed the widest variability, reflecting dependency on farm conditions and the definition of estrus events. Physiological sensors tended to yield higher consistency but were tested on limited datasets, reducing generalizability. The scarcity of studies reporting full accuracy metrics further complicates direct comparison. These findings reinforce a key challenge also seen in mastitis detection: most sensor systems still show “less-than-perfect” performance, which limits their reliability for autonomous decision support at the farm level. Consistency across farms and real-world validations remains essential steps toward achieving Level III or IV.

On locomotion sensor systems, reported sensitivities typically ranged from 58% to 97%, while specificities were often higher, between 42% and 99%. Only a few camera- or pressure-based systems achieved both metrics above 95%. Accelerometer-based sensors and indoor positioning systems provided consistent but conservative results, high specificity yet lower sensitivity, reflecting their reliance on indirect behavioral indicators such as reduced activity or fewer feeding visits. Vision-based systems using deep learning models (e.g., convolutional neural network) reached higher accuracy but required controlled lighting and camera angles, limiting robustness under farm conditions. Force-plate systems demonstrated the most precise asymmetrical detection in experimental setups, though they remain impractical for routine farm use due to cost and cow throughput constraints. These results underline the persistent challenge of “not-perfectly-performing” locomotion sensors: while algorithms are increasingly sophisticated, variability in barn environment, surface type, and cow posture reduces repeatability. Future work should aim for integrated multimodal solutions by combining motion, pressure, and behavioral data to improve early detection reliability and enable practical deployment in commercial dairy operations.

Meanwhile, for metabolism disorder detection, the reviewed sensor systems generally showed sensitivity ranging between 57% and 93% and hardly any specificities were provided. These values indicate that sensors can capture physiological changes such as alterations in rumination time, milk composition, or body temperature. The results likely arise from the complex, multifactorial nature of metabolic disorders, where physiological indicators overlap with normal postpartum variations. To improve robustness, future research should combine multi-sensor data streams with longitudinal validation across herds to better capture individual variability and improve detection reliability.

## ADOPTION OF PRECISION DAIRY FARMING

In a certain way, the results of this review confirm this disappointing development of adoption of PDF. Research is still focused on detection algorithms, while in the evolution of research one would expect more work on the integration and automation levels. The far majority of articles are describing novel algorithms or sensors and seem to be struggling to improve the performance of sensor systems. Papers on the decision support that can be done and the effects of use are almost non-existent. This review’s classification relied on published descriptions; systems may have undocumented integration features. We excluded gray literature and commercial product reports. It is possible that Level III and IV functionalities exist in proprietary farm commercial software without scientifically published papers. Additionally, some sensor systems might have integration modules currently under development and have not yet been documented in scientific articles. Therefore, scientific research may be outdated compared to real-world application in commercial/practical farms. Despite these considerations, the authors do think that relatively little is done on how to create value with sensor systems. Research should be more focused on improved management with the current sensor systems, and take the current performance level of sensor systems for granted. As information alone does not have much value, we need to combine this information with tailormade interventions. Some ideas for improved mastitis management have been worked out conceptually [47]. That way, sensor-derived information, combined with tailored interventions can create value [53].

Associated with the adoption issue, the cost-effectiveness of investment in sensors is crucial. Consequently, it is not sufficient to evaluate a sensor systems only sensitivity and specificity. For a sensor technology to be viable, the economic impact of its use must be quantified. Farmers, typically, make investment decisions based on return on investment [7], so without concrete figures on cost savings or revenue gains, even the most accurate detection algorithms risk remaining limited to just research output. For example, an early mastitis detection system could report the average reduction in antibiotic treatments per cow per year or the associated treatment cost savings. Similarly, fertility detection tools might quantify increased conception rates, reduced days open, and additional kilograms of milk produced per lactation. Labor savings, measured in hours of manual herd inspection avoided, can be translated into wage cost reductions or redeployment of personnel to higher-value tasks. By presenting this financial calculation together with sensitivity and specificity, researchers can demonstrate a clear business case for sensor system adoption.

This can be done in early stage sensor system research, by developing simulation models that show the economic consequences with various sensor system performance in combination with different interventions, as was recently done for lameness detection [48]. Going forward, research must move beyond model accuracy to co–develop solutions with software providers and integrate workflows that show farmers exactly how sensor insights translate into advice such as saving labor costs, reducing disease costs, or increasing milk revenue. Only then will precision dairy technologies achieve practical uptake for farmers.

## CONCLUSIONS

This review shows that, despite substantial activity in sensor development for PDF, most published systems remain focused on data interpretation rather than integrated decision support or providing fully automated, actionable decision support. Crucially, almost no published sensor system in the reviewed articles quantifies economic benefits, leaving return-on-investment of sensor system adoption unproven. To move from proof-of-concept towards practical implementation, future research must integrate sensor outputs with herd records and financial models, validate systems across multiple farms and production systems, and operate at higher data frequencies. Farmer-centered design, including workflow integration and mechanisms to prevent alert fatigue, is essential to ensure that technical accuracy translates into effective on-farm action. Economic evaluation should become a standard part of system validation, as accuracy alone does not guarantee practical value or widespread adoption.

## Figures and Tables

**Table 1:** The query of searching on database resource

Parameters	Details
Database	Scopus
Search strings	sensor* AND dair* AND farm* AND automat* AND detect* AND monitor* AND prediction* AND (mastitis OR locomotion OR lameness OR metabolic OR estrus OR fertility) AND PUBYEAR > 2013 AND PUBYEAR < 2025 AND (EXCLUDE (PUBSTAGE, “aip”) AND (LIMIT-TO (LANGUAGE, “English”)
Type of documents	Journal articles and conference proceedings
Year	2014–2024
Language	English
Excluded keywords	“Beef Cattle”, “Crops”, “Pig”, “Poultry”, “Sheep”
Results	561

**Table 2:** Summary table of sensors for mastitis detection in dairy cows

Dev. level	Articles	Sensor type	GS	Alg.	Se. (%)	Sp. (%)	Acc. (%)
Level I	He et al [20]	Optical sensor	SCC	N/A	79	N/A	98
	Gayathri et al [21]	IR camera	N/A	MLM	N/A	N/A	N/A
	Sathiyabarathi et al [22]	IR thermal camera	N/A	N/A	N/A	N/A	N/A
Level II	Fan et al [24]	EC, SCC, milk composition data, milk yield	SCC	LogReg, SVM, NB, DT	82–95	93–95	N/A
	Liebe et al [25]	Multi-sensor suite (leg, ear, neck tags; bolus; milking meter)	N/A	GBT, XGBoost	37.5–52.63	99.78–99.85	N/A
	Dutta et al [41]	Activity sensor (temperature sensor, GPS module, 3D accelerometer)	N/A	XGBoost, Cl.	97	98	98
	Jensen et al [54]	EC, SCC, milk yield	SCC	Multivariate DLM, NBC	80	81	N/A
	Lien et al [55]	EC	N/A	SR	46.2	83.7	90.8
	Jensen et al [56]	EC, SCC	SCC	EM, DLM	N/A	N/A	95
	Khatun et al [57]	EC, SCC, milk volume	SCC	GLMM	N/A	N/A	N/A
	Paudyal et al [58]	SCC	SCC	Predictive models and SA	42–80	84	-
	Ebrahimi et al [59]	EC, activity, rumination	N/A	DL, GBT, DT, GLM, LM, LogReg, NB, RF	93–99	1.2–39.7	84.9
	Khatun et al [60]	Milk yield, milk component level	N/A	GLMM	89.2–96.2	88–94.9	N/A
	Post et al [61]	EC, SCC, milk volume	N/A	LogReg, SVM, kNN, GNB, ETC, RF	79	79	N/A
	Steele et al [62]	EC, SCC, LDH activity, milk protein, and fat	SCC	MLR	N/A	82	94
	Anglart et al [63]	EC, milk yield, duration, milk flow	N/A	GAM, DT, ANN - MLP	N/A	N/A	N/A
	Ebrahimie et al [64]	Daily milk component & activity sensors	Bacteriological culture (gram-negative/Gram-positive/NPI)	CBA	N/A	N/A	-
	Post et al [65]	CMSCC	SCC	Cl.RGtreat-SC/PL, PPV	72–87	42–63	N/A
	Robles et al [66]	OCC	SCC	SA	44–86	N/A	93–96
	Tsai et al [67]	EC, milk volume, fat, protein, lactose, milking time, and milking peak flow	SCC	GLM	N/A	N/A	N/A
	Bausewein et al [68]	Milk yield, duration, milk flow, EC	N/A	GLMM	31–78	79–97	N/A
	Bonestroo et al [69]	SCC, TLC	Flow Cytometry	GBT, XGBoost	N/A	N/A	55.2–88.8
	Naqvi et al [70]	EC, milk yield, blood occurrence	SCC	RNN	76–86	85–90	N/A
	Naqvi et al [71]	OCC, EC	SCC	RNN, LSTM	77.9–86.3	78.1–82.4	N/A
	Yang et al [72]	AMS milking sensor	SCC	DRPs-NNUSVR	N/A	N/A	N/A
	Cho et al [73]	AMS milking sensor	N/A	Cl., ANOVA	44–65	94–98	93–97
	D’Anvers et al [74]	EC, milk yield (VMS)	N/A	IG, PCR	N/A	N/A	46–57
	Faris et al [75]	In-line sensor	N/A	SVM, LogReg, GNB, kNN, CART-DT, RF	N/A	N/A	62–74
	Jin et al [76]	EC, milk yield	N/A	*k*NN, ROCKET, HIVE-COTE 2.0, LogReg, NB, GBT	N/A	N/A	79.3
	Olofsson et al [77]	EC, milk yield	N/A	LinReg	N/A	N/A	N/A
	Anglart et al [19]	AMS milking sensor	N/A	MLP, ANN	26	98–100	N/A
	Khatun et al [78]	EC, SCC, milk yield	SCC	DT	40–66.7	99	N/A
	Ebrahimie et al [79]	SCC, EC, milk yield, AMF	N/A	AWM	89	92	50
	Xudong et al [80]	FLIR	Historical veterinary records	EFMYOLOv3	N/A	92.31	76.47
	Ramirez-Morales et al [81]	NIR	N/A	kNN	85.8–95	94–97.7	92.4–95
	Wang et al [82]	FLIR	N/A	LogReg	89.4	N/A	N/A
	Zhang et al [23]	FLIR	N/A	CLE-UNet	N/A	82.35	80
	Wang et al [83]	FLIR	N/A	DCYOLO	79	92	N/A
	Khatun et al [84]	Neck-mounted tag & in-line EC sensor	N/A	GLMM	87.8	89.5	N/A
	Feng et al [85]	GPS	SCC	SNA	N/A	28	N/A
	Grodkowski et al [86]	3D Motion Detector	N/A	ANN, LogReg	74–79	76–79	N/A
	Hu et al [87]	Smart dairy farm sensing network	SCC	Clustering analysis and ANOVA	76.3	70.2	88.91
	Antanaitis et al [88]	Reticulorumen temperature (RR temp.), reticulorumen pH (RR pH), and cow activity	SCC	SA, ANOVA	N/A	N/A	N/A
	Kuma et al [89]	Flex sensors and a temperature sensor	SCC	RF, SVM, kNN, DT, NB	N/A	N/A	99.1–99.46
	Rodriguez et al [90]	Reticuloruminal sensor (bolus)	Bacterial culture	Reg.	70	86.7	90.9
	Vidal et al [91]	Ear-tag accelerometer	N/A	RF	N/A	95.12	100
	Zhou et al [92]	Neck collar	N/A	LogReg	71.05	N/A	N/A
	Khan et al [93]	3D accelerometer, behavioural sensor	N/A	SVM	87.5–88.5	88.7–90.4	88.1–89.4
	Nadeem et al [94]	Activity sensor	N/A	RF, XGBoost, LogReg, SLP	100	N/A	98.25
	Santos et al [95]	Neck collar	N/A	Cl.	N/A	43	81
	Tian et al [96]	Neck collars	N/A	MNET	N/A	91.89	93
Level III	Saro et al [26]	Integration between sensor system and historical veterinary records	Multiple sensor combined with historical veterinary records (disease incidence data)	LSTM	N/A	N/A	N/A

Dev. level, development level; GS, type of gold standard; Alg., algorithm; Se., sensitivity; Sp., specificity; Acc., accuracy; SCC, somatic cell count; N/A, not available; IR, infrared; MLM, multivariate linear model; EC, electrical conductivity; NIR, near infrared; OCC, online cell counter; CMSCC, comparison of methods for predicting cow composite somatic cell counts; TLC, total leukocyte count; AMF, average milk flowrate; FLIR, forward looking infrared camera; ETC, extra trees classifier; DLM, dynamic linear model; NBC, naïve bayesian classifier; MLP, multi-layer perceptron; ANN, artificial neural network; DT, decision tree; AWM, attribute weighting algorithm; EM, expectation-maximization; DL, deep learning; GBT, gradient-boosted trees; LinReg, linear regression; LogReg, logistic regression; kNN, k-nearest neighbors; GNB, gaussian naïve bayes; NB, naïve bayes, ET, extra trees classifier; MLR, multivariate logistic regression; GAM, generalized additive model; CBA, classification-based algorithm; PPV, positive predictive value; SA, statistical analysis; GLM, generalized linear model; GLMM, generalized linear mixed model (GLMM); XGBoost, extreme gradient boosting; RNN, recurrent neural networks; DRPs-NNUSVR, nonlinear nu-support vector regression with dielectric relaxation parameters; ANOVA, analysis of variance; IG, incomplete gamma lactation curve model; PCR, principal component regression; CART-DT, classification and regression decision tree; ROCKET, random convolutional kernel transform; HIVE-COTE 2.0, hierarchical vote collective of transformation-based ensembles; GBM, gradient boosting machine; EFMYOLOv3, enhanced fusion mobilenetv3 you only look once v3; DCYOLO, DeepLabV3+ and CAYOLOV5; SNA, social network analysis; KS, kolmogorov-smirnov; SLP, single layer perceptron; LSTM, long short-term memory; MNET, multilayer artificial neural net; RF, random forest; Reg., regression; Cl., classification; SVM, support vector machine.

**Table 3:** Summary table of sensors for fertility detection in dairy cows

Dev. level	Articles	Sensor type	GS	Alg.	Se. (%)	Sp. (%)	Acc. (%)
Level I	Röttgen et al [27]	Microphone	Video-observed vocalizations	87	94	N/A	N/A
	Antanaitis et al [28]	Accelerometer, pressure sensor	N/A	N/A	N/A	N/A	N/A
	Mozūraitis et al [29]	Ultrasound	N/A	N/A	N/A	N/A	N/A
	Eckelkamp et al [30]	3D accelerometer	N/A	N/A	N/A	N/A	N/A
	Ren et al [31]	Accelerometer, temperature sensor	N/A	N/A	N/A	N/A	N/A
	Arago et al [32]	Video cameras	N/A	N/A	N/A	N/A	N/A
	Pfeiffer et al [33]	Camera, rumen bolus sensor	Analysis by trained observer	N/A	N/A	N/A	N/A
Level II	Hut et al [34]	Neck-collar, leg tag	N/A	MLME	N/A	N/A	88–97
	Mayo et al [35]	Pedometer, bolus, neck collar, ear tag, temperature sensor	Standing estrus (behavioral)	PA	41.8–92.2	N/A	40–100
	Pereira et al [36]	Activity and rumination monitoring sensor	N/A	SA	56.7–72.5	99.2–99.3	N/A
	Dutta et al [41]	Activity sensor, 3D accelerometer	N/A	XGBoost	97.1	98.1	97
	Paudyal et al [58]	Neck collar rumination loggers	N/A	Mates index (MIx) alert model	63	84.6	N/A
	Tsai et al [67]	Accelerometer, DVM bolus, milk meters	N/A	GLM	N/A	N/A	N/A
	Vidal et al [91]	Accelerometer	N/A	Threshold based on blood/clinical test	92.5–100	95–100	97.8–99.5
	Nadeem et al [94]	Activity sensor	N/A	RF	100	N/A	91.60
	Reith et al [97]	Accelerometer, electronic floor scale	N/A	SA	N/A	N/A	N/A
	Silper et al [98]	Ultrasound, accelerometer, milk production	Walking activity	MLR	78.6–82.5	34.3–40.1	N/A
	Brassel et al [99]	Accelerometer	N/A	SA	93.3	N/A	N/A
	Hamilton et al [100]	Accelerometer	N/A	SVM	89.2	88.8	N/A
	Schweinzet al [101]	Accelerometer	N/A	ML	96.6–96.8;	97.7	N/A
	Lovarelli et al [102]	Accelerometer	N/A	PCA, FA, GLMSelect, LA, CA	N/A	N/A	N/A
	Belaid et al [103]	Accelerometer	N/A	GLIMMIX	80	58.1	59.1
	Lopreiato et al [104]	Neck Collar	N/A	kMC	N/A	N/A	N/A
	Macmillan et al [105]	Ear tag	N/A	GLIMMIX, ANOVA	N/A	N/A	N/A
	Rachah et al [106]	FTIR	N/A	PCA, PLSR, SPLSDA	N/A	N/A	N/A
	Schilkowsky et al [107]	Accelerometer	RTE, Visual Observation,	LogReg, ANOVA, PROC MIXED	89.6–92.5	69.2–91.7	88–94
	Fadul et al [108]	Noseband, pedometer	N/A	UVA, MVA, LogReg	73.7	82.4	81
	Higaki et al [109]	Accelerometer, Thermistor	N/A	SVM, EWMA	90.5–97	77.9–89.1	N/A
	Wang et al [110]	Accelerometer	N/A	MMDC	83.3	N/A	100
	Wang et al [111]	Accelerometer	N/A	BPNN, SVM, LogReg	95.84–98.78	91.07–95.08	91.98–95.46
	Zhou et al [112]	Activity, rumination, milking sensor	N/A	DT, SVM, RF, XGBoost, Adaboost, NB, kNN, LogReg	83.33	N/A	84.21
	Arıkan et al [113]	Camera	N/A	CNN, VGG-19, YOLO	98	N/A	N/A
	Cook [114]	Eartag sensor, activity sensor, temperature sensor	N/A	Multivariable LogReg	42.4	75.2	62.5
	Jia et al [115]	Camera	N/A	CAMLLA-YOLOv8n	94.16	N/A	94.04–92.97
	Leliveld et al [116]	Accelerometer	N/A	GLIMMIX	N/A	N/A	N/A
	Lodkaew et al [117]	Camera	N/A	YOLOv4, CNN, LightGBM, XGBoost, CatBoost, LightGBM	79–81	N/A	83
	Zhou et al [118]	Neck collar	N/A	ANOVA, LogReg	N/A	N/A	N/A
	Khin et al [119]	Accelerometer	N/A	Catboost	96	95.8	95.9
	Li et al [120]	Camera	N/A	IATEFF-YOLO	99.4	N/A	99.1
	Mladenova et al [121]	Accelerometer, gyroscope	N/A	RFE, DT, SVM, NB	N/A	N/A	99
	Rial et al [122]	Neck collar, AMS milking sensor	N/A	LogReg, ANOVA, Poi.	N/A	N/A	N/A
	Shokrollahi et al [123]	IR thermal sensor	N/A	LM	N/A	N/A	N/A
	Adriaens et al [124]	AMS milking sensor	N/A	ANOVA	N/A	N/A	N/A
	Higaki et al [125]	Vaginal sensor (thermistor and ring conductivity electrodes)	N/A	EWMA, ANN, SVM, DT	94	N/A	N/A
	Vicentini et al [126]	Digital Thermometer (rectal and vaginal), thermal imaging camera	N/A	SA	N/A	N/A	N/A
	Huot et al [127]	Wireless bolus sensors	N/A	MLM	N/A	N/A	N/A
	Risvanli et al [128]	Metrisor (gas sensor)	N/A	ICO, RF	71.2–78.2	N/A	71.2–78.16
	Chin et al [129]	BHB sensor	N/A	Cox.	N/A	N/A	N/A
	Benaissa et al [130]	UWB localization sensor; accelerometer	Visual observation	LogReg, kNN	85–90	98–99	N/A
	Cairo et al [131]	Electronic feed bins and water bins	Visual observation	ANN, RF, GLM	98.8–100	86.4–93.2	93.0–96.5

Dev. level, development level; GS, type of gold standard; Alg., algorithm; Se., sensitivity; Sp., specificity; Acc., accuracy; N/A, not available; RTE, reference test for estrus; MVA, multivariate analysis; UVA, univariate analysis; MIx, mates index alert model; GLM, generalized linear model; XGBoost, extreme gradient boosting; RF, random forest; SA, statistical analysis; MLR, multivariate logistic regression; SVM, support vector machine; ML, machine learning; PCA, principal components analysis; FA, factor analysis, GLMSelect, generalized linear model select; LA, logistic analysis; CA, cluster analysis; GLIMMIX, generalized linear mixed model with PROC GLIMMIX; RTE, reference test for estrus; kMC, k-means clustering; PLSR, partial least squares regression, FTIR, Fourier transform infrared spectroscopy; SPLSDA, sparse partial least squares discriminant analysis; PLSDA, partial least squares discriminant analysis; ANOVA, analysis of variance; LogReg, logistic regression; ROC, receiver operating characteristic; EWMA, exponentially weighted moving average; MLME, multivariable linear mixed effect models; MMDC, maximum-minimum distance clustering; BPPN, backpropagation neural network; DT, decision tree; CAMLLA-YOLOv8n, Improved YOLOv8n with C2f-CA MLLAttention, SPPF-GPE, and shape-IoU loss; YOLOv4, you only look once version 4; CNN, convolutional neural network; LightGBM, light gradient-boosting machine; GBM, gradient boosting machine; CatBoost, gradient boosting on decision trees; IATEFF-YOLO, illumination adaptive transformer EFF-YOLO; RFE, random forest ensemble; NBC, naïve bayesian classifier; LogReg, logistic regression; Poi., poisson regression; LMM, linear mixed models; ANN, artificial neural network; ICO, iterative classifier optimizer; Cox., cox proportional hazard regression models; kNN, k-nearest neighbors, CZT, chirplet z transform; CDF, cumulative distribution frequency; CART, classification and regression tree; MST, minimum spanning tree; ACH, approximate convex hull; NCBoP, non-convex boundary over projections; PART, partial tree; UWB, ultra-wide band.

**Table 4:** Summary table of sensors for locomotion problem detection in dairy cows

Dev. level	Articles	Sensor type	GS	Alg.	Se. (%)	Sp. (%)	Acc. (%)
Level I	Antanaitis et al [28]	Noseband sensor, accelerometer	N/A	SA, LinReg, ANOVA, PCC.	N/A	N/A	N/A
	Schodl et al [37]	3D accelerometer	N/A	PCA, ANN, BN	N/A	N/A	N/A
Level II	Balasso et al [38]	3D accelerometer	N/A	CNN, RF, kNN, XGBoost, SVM	93–99	N/A	96
	Beer et al [39]	3D accelerometer	Gait	UVA, MVA, ROC-analysis	90.2	91.7	N/A
	Borghart et al [40]	3D accelerometer, live weight sensor	LMS	XGBoost	85	78	N/A
	Dutta et al [41]	3D accelerometer, temperature sensor, GPS	Manual observation	XGBoost, RF	75–100	N/A	N/A
	Zhang et al [42]	3D accelerometer, gyroscope	NRS	Semi-supervised LSTM-autoencoder	96.67	98.33	N/A
	Paudyal et al [58]	Rumination loggers	N/A	SA	42–100	84–85	N/A
	Post et al [61]	Pedometer	N/A	LogReg, SVM, kNN, GNB, DT, RF	71	66	N/A
	Post et al [65]	Pedometer	N/A	CART, GBM, XGBoost, RF	71	42	N/A
	Vidal et al [91]	3D accelerometer	LMS	RF, k-NN, SVM	94	98	98
	Zhou et al [92]	Neck collar and pedometer	Clinical diagnoses by veterinarians and farm staff	LogReg	63.16	96.51	90.48
	Nadeem et al [94]	Cow activity	N/A	RF, XGBoost, LogReg, SLP	71	42	91.47
	Fadul et al [108]	Noseband sensor	Clinical diagnosis by expert clinicians (lab analysis, BHB, glucose, blood gas)	UVA, MVA	73.7	88.2	N/A
	Khin et al [119]	Camera	N/A	Mask R-CNN, YOLOv8	N/A	N/A	N/A
	Mladenova et al [121]	3D accelerometer, gyroscope	N/A	RFE, DT, SVM. NB	N/A	N/A	99
	Gusterer et al [132]	3D accelerometer	N/A	ANOVA	N/A	N/A	N/A
	Frondelius et al [133]	Indoor positioning system, RIC	LMS	LMM	N/A	N/A	N/A
	Brouwers et al [134]	Accelerometer	LMS	DL, HIVE-COTE 2.0	N/A	N/A	74
	Ismail et al [135]	3D accelerometer, gyroscope, magnetometer	LMS	SVM (using CZT and CDF)	71–83	N/A	77
	Neupane et al [136]	3D acceleromter	LMS	ROCKET, RF, NB, LinReg	60–84	94–99	90–93
	Peng et al [137]	Accelerometer, gyroscope, magnetometer	Manual labeling by experts	Improved EdgeNeXt (DL), Swin Transformer, MobileNetV2, ConvNeXt, EdgeNeXt, EdgeNeXt-BNHS	N/A	N/A	95.85–97.6
	Russel et al [138]	3D accelerometer	Video recordings (human observers labeling behaviors)	CNN-based 23-layer framework	N/A	N/A	87.1–98.7
	Van Hertem et al [139]	3D depth camera	LMS	Image processing	N/A	N/A	N/A
	Piette et al [140]	3D camera	LMS	Deviation detection algorithm	79	82.3	82
	Warner et al [141]	Video camera	N/A	CART, GBM, XGBoost, RF, GLM	58	89	N/A
	Brezov et al [142]	FLIR	N/A	AutoGluon (NeuralNetTorch, XGBoost, CatBoost)	N/A	N/A	N/A
	Hua et al [143]	Video camera	Expert standards	YOLOX-Pose	N/A	N/A	92.6
	Mon et al [144]	RGB image-based camera	Manual identification	YOLOv8, VGG16, SVM	N/A	N/A	96.34
	Garcia et al [145]	AMS sensor	N/A	PLS-DA	79	83	N/A
	Grimm et al [146]	Pedometer, AMS sensor	Weekly LMS	ENET, ENET beta, LogReg	94	81	N/A
	Lemmens et al [147]	Ear/collar tags, AMS sensor	LMS, BCS	RF	72	78	75
	Jukna et al [148]	Milk and blood sensor	LS	LinReg, multivariable binary LogReg	N/A	N/A	N/A
	Michelena et al [149]	Accelerometer, BCS	N/A	PCA, kMC, kNN, MST ACH, NCBoP	N/A	N/A	N/A
	Swain et al [150]	Multi sensor (feed intake monitors, motion sensors, blood oxygen sensors, estrus monitors, sound sensors, respiration rate monitors, weight sensors, imaging sensors)	N/A	Ensemble learning (RF+CatBoost), NBM, lazy-IBk, PART, SVM	N/A	N/A	88

Dev. level, development level; GS, type of gold standard; Alg., algorithm; Se., sensitivity; Sp., specificity; Acc., accuracy; N/A, not available; BCS, body condition score, LMS, locomotion score; LS, lameness score; Gait, gait score; CD, clinical diagnosis; NRS, numerical rating system; BHB, β-hydroxybutyrate; RIC, roughage intake control; SA, statistical analysis; LinReg, linear regression; LogReg, logistic regression; ANOVA, analysis of variance; PCC, pearson correlation coefficient; PCA, principal component analysis; ANN, artificial neural network; BN, bayesian network; MVA, multivariate analysis; UVA, univariate analysis; ROC, Receiver Operating Characteristic; XGBoost, extreme gradient boosting; RF, random forest; CNN, convolutional neural network; kNN, k-nearest neighbors; SVM, support vector machine; DL, deep learning, LSTM, long short term memory; CZT, chirplet z transform, CDF, cumulative distribution frequency; ROCKET, random convolutions kernel transformation; NB, naïve bayes; CART, classification and regression tree; GBM, gradient boosting machine; MML, multimodal machine learning; PLS-DA, partial least squares discriminant analysis; ENET, elastic net; GNB, gaussian naïve bayes; DT, decision tree; MST, minimum spanning tree; ACH, approximate convex hull; NCBoP, non-convex boundary over projections; SLP, single layer perceptron; NBM, naïve bayes multinomial.

**Table 5:** Summary table of sensors for metabolism problem detection in dairy cows

Dev. level	Articles	Sensor type	GS	Alg.	Se. (%)	Sp. (%)	Acc. (%)
Level II	Ferreira et al [43]	Wearable sensors; imaging systems	BHB	Multi-modal ML; UMAP; ResNet-50; LASSO	N/A	N/A	N/A
	Heirbaut et al [44]	Mid-infrared spectroscopy; camera; accelerometer	Blood metabolites	kMC, LM, SPLS	N/A	N/A	N/A
	Taechachokevivat et al [45]	In-line milk sensor	CD	LSTM	62–83	N/A	N/A
	Simoni et al [46]	Accelerometer	CD	PA for health alerts (based on rumination time), mdixed ANOVA	71	47	N/A
	Lopreiato et al [104]	Activity sensor	BHB, NEFA, haptoglobin, and CD of disorders	kMC	N/A	N/A	N/A
	Rachah et al [106]	FTIR	BHB	PCA; PLSR; SPLSDA	N/A	N/A	N/A
	Antanaitis et al [151]	In-line milk sensor	Clinical signs and milk FPR	PA for HIS, LogReg	93	N/A	N/A
	Giannuzzi et al [152]	In-line milk sensor	Reference blood metabolic profile analysis	Multi-layer feedforward ANN; penalized regression (RR, LASSO, EN); RF; GBM	N/A	N/A	N/A
	Satoła et al [153]	In-line milk sensor	BHB	LogReg, SVC, RFE	72–74	N/A	80–81
	Antanaitis et al [154]	Activity sensor, pedometer	BHB	PCC	N/A	N/A	N/A
	Rial et al [155]	Accelerometer	CD	LMM	N/A	N/A	N/A
	Stangaferro et al [156]	Activity sensor	CD	SA	93	N/A	N/A
	Heirbaut et al [157]	Reticuloruminal pH boluses, activity sensor; eating/rumination sensor	Blood metabolites	kMC, DLM. LME, RF	N/A	N/A	N/A
Level III	Saro et al [26]	Historical veterinary records on disease incidence, treatments, costs	N/A	LSTM	N/A	N/A	2.35–6.66

Dev. level, development level; GS, type of gold standard; Alg., algorithm; Se., sensitivity; Sp., specificity; Acc., accuracy; N/A, not available; BHB, β-hydroxybutyrate; FPR, fat-protein ratio; CD, clinical diagnosis; SOP, standard operational procedure; SCK, subclinical ketosis; NEFA, non-esterified fatty acids; IGF-1, insulin-like growth factor 1; SCA, subclinical acidosis; HIS, health index score; LogReg, logistic regression; MFFNN, multilayer feed-forward neural network; RR, ridge regression; LASSO, least absolute shrinkage and selection operator; EN, elastic net; RF, random forest; GBM, gradient boosting machine; SVC, support vector classification; RFE, recursive feature elimination; LSTM, long short-term memory; PCA, principal component analysis; PLSR, partial least squares regression; SPLSDA, sparse partial least squares discriminant analysis; PCC, pearson correlation coefficient; kMC, k-means clustering; LMM, linear mixed models; Mixed ANOVA, mixed-design analysis of variance; PA, proprietary algorithms; SA, statistical analysis; CD, clinical Diagnosis SPLS, sparse partial least squares; DLM, dynamic linear model; LME, linear mixed effect models; MML, multi-modal machine learning; UMAP, uniform manifold approximation and projection; ResNet-50, 50-layer deep convolutional neural network; LSTM, long short-term memory; kMC, k-means clustering.

## Data Availability

Upon reasonable request, the datasets of this study can be available from the corresponding author.
